# Current treatment and novel insights regarding ROS1‐targeted therapy in malignant tumors

**DOI:** 10.1002/cam4.7201

**Published:** 2024-04-17

**Authors:** Shizhe Li, He Zhang, Ting Chen, Xiaowen Zhang, Guanning Shang

**Affiliations:** ^1^ Department of Orthopedics Shengjing Hospital of China Medical University Shenyang Liaoning Province China; ^2^ Medical Research Center Shengjing Hospital of China Medical University Shenyang Liaoning Province China

**Keywords:** gene fusion, ROS1 gene, tumor‐targeted therapy, tyrosine kinase inhibitor

## Abstract

**Background:**

The proto‐oncogene ROS1 encodes an intrinsic type I membrane protein of the tyrosine kinase/insulin receptor family. ROS1 facilitates the progression of various malignancies via self‐mutations or rearrangements. Studies on ROS1‐directed tyrosine kinase inhibitors have been conducted, and some have been approved by the FDA for clinical use. However, the adverse effects and mechanisms of resistance associated with ROS1 inhibitors remain unknown. In addition, next‐generation ROS1 inhibitors, which have the advantage of treating central nervous system metastases and alleviating endogenous drug resistance, are still in the clinical trial stage.

**Method:**

In this study, we searched relevant articles reporting the mechanism and clinical application of ROS1 in recent years; systematically reviewed the biological mechanisms, diagnostic methods, and research progress on ROS1 inhibitors; and provided perspectives for the future of ROS1‐targeted therapy.

**Results:**

ROS1 is most expressed in malignant tumours. Only a few ROS1 kinase inhibitors are currently approved for use in NSCLC, the efficacy of other TKIs for NSCLC and other malignancies has not been ascertained. There is no effective standard treatment for adverse events or resistance to ROS1‐targeted therapy. Next‐generation TKIs appear capable of overcoming resistance and delaying central nervous system metastasis, but with a greater incidence of adverse effects.

**Conclusions:**

Further research on next‐generation TKIs regarding the localization of ROS1 and its fusion partners, binding sites for targeted drugs, and coadministration with other drugs is required. The correlation between TKIs and chemotherapy or immunotherapy in clinical practice requires further study.

## Graphical abstract

This study searched for relevant articles on the mechanism and clinical application of the ROS1 gene in recent years, systematically reviewed the biological mechanisms, diagnostic methods, and research progress on ROS1 inhibitors, and prospected the future of ROS1 targeted therapy to provide new ideas for clinical application and treatment. We believe that our study makes a significant contribution to the literature because our review revealed the following findings: the proto‐oncogene ROS1 is mostly expressed in malignant tumours, such as non‐small cell lung cancer (NSCLC). Only a few ROS1 kinase inhibitors are currently approved for use in NSCLC. At the same time, the efficacy of other tyrosine kinase inhibitors (TKIs) for NSCLC and other malignancies has not been ascertained as they are still being tested in clinical trials. There is no standard effective treatment for adverse events or resistance to ROS1‐targeted therapy. Finally, next‐generation TKIs appear capable of overcoming resistance and delaying central nervous system metastasis owing to their high affinity, but with a greater incidence of adverse effects.

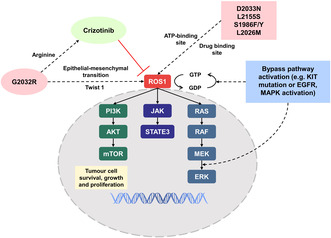

## 1 INTRODUCTION

The proto‐oncogene ROS1 (c‐ROS) was first identified in glioblastoma cells in 1987^1^ and is located at position 6q22 on the long arm of chromosome 6.^2^ The ROS1 protein encoded by the *ROS1* gene is a type I intrinsic transmembrane tyrosine kinase receptor belonging to the insulin receptor family.^3^ The self‐mutation or rearrangement of ROS1 can activate tyrosine kinase, which promotes the proliferation of various malignant tumors.^4^ ROS1 was subsequently detected in non‐small cell lung cancer (NSCLC) in 2007 as a chromosomal rearrangement^5^ and contains fusion genes, such as SLC34A2‐ROS1,^6^ SDC4‐ROS1, and CD74‐ROS1.^7^ The fusion of ROS1 can lead to autophosphorylation, mediating tumor progression through the mitogen‐activated protein kinase (MAPK) pathway or RAS phosphorylation.^8^, ^9^

Clinical trials of ROS1‐directed tyrosine kinase inhibitors (TKIs) have been conducted, showing considerable results. Currently, several ROS1‐targeted drugs, such as crizotinib^10^ and ceritinib,^11^ have been approved by the FDA for use in a variety of malignancies, especially ROS1‐positive NSCLC.^3^ Although progress has been made with the use of ROS1 inhibitors in clinical practice, presistant challenges such as drug resistance and adverse reactions (including central nervous system [CNS] metastases) remain unresolved. Remon et al.^12^ suggested that next‐generation TKIs with high affinity and selectivity could be effective against drug resistance and CNS metastasis in ROS1‐targeted therapy. However, these drugs have not been utilized in clinical practice, as they are still undergoing clinical trials. Therefore, elucidation of the biological mechanism of the *ROS1* gene, including the mechanism of *ROS1* gene mutation, clarification of the concept of targeted therapy, and development of next‐generation inhibitors are crucial. In this study, ROS1‐related articles were retrieved from PubMed/MEDLINE and Web of Science (search keywords: “ROS1” and “malignant tumors”) to review the biological mechanisms, diagnostic modalities, and treatment effectiveness of ROS1 inhibitors to provide new insights into their clinical applications, therapeutic strategies, and directions for future development.

## 2 BIOLOGICAL MECHANISMS OF THE *ROS1* GENE

### 2.1 Nonmalignant biological mechanisms of the *ROS1* gene

**FIGURE 1:**
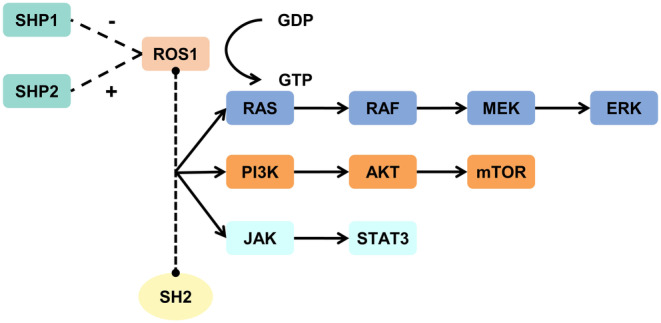
Nonmalignant biological signaling pathways involving the *ROS1* gene.

ROS1 contains 47 exons, of which exons 1–34 encode the largest extracellular N‐terminal domain of the human receptor tyrosine family.^13^ Springer et al.^14^ found that this domain is composed of three YWTD domains and scattered type III fibronectin (FN3). The YWTD domain has a round and folded “propeller” shape, which can closely interact with surrounding structures, and change the structure of ROS1. In addition, ROS1 and the anaplastic lymphoma kinase (ALK) domains are very similar at the amino acid level, sharing 77% identity at the ATP‐binding site.^15^ Considering the similarity between ROS1 and ALK, ALK inhibitors are also commonly used in ROS1‐mutant tumors.^16^ The signaling pathways involving ROS1 are related to the differentiation and proliferation of normal cells. ROS1 can activate and bind to the SH2 domain through the intracellular autophosphorylation of specific tyrosines, acting with specific adaptor proteins to mediate the RAS/RAF/MEK/ERK,^17^ PI3K/AKT/mTOR,^18^ and JAK/STAT3^19^ signaling pathways (Figure 1). ROS1 can activate both PTPN6 (SHP1) and PTPN11 (SHP2). Despite their structural homology, PTPN6 often inhibits cell activity through a negative regulatory pathway, whereas PTPN11 mainly acts as a positive signal transducer.^20^, ^21^

### 2.2 Malignant biological mechanisms of the *ROS1* gene

ROS1 can facilitate the progression of various tumors through its overexpression, mutation amplification, or gene fusion. The overexpression of ROS1 was initially discovered in neurological tumors, such as primary gliomas^22^ and meningiomas.^23^ Cheng et al.^24^ found that *ROS1* gene expression in the cytoplasm was positively correlated with the development of oral squamous cell carcinoma (OSCC). It has been suggested that ROS1 can mediate the invasiveness of OSCC by enhancing mitochondrial bioactivity and increasing cellular ATP levels.^25^ Boyard et al.^26^ found that the rearrangement and overexpression of ROS1 can activate the JAK/STAT pathway in inflammatory hepatocellular adenoma and promote its progression. Bajrami et al. suggested that ROS1 is highly expressed in E‐cadherin (CDH1)‐deficient breast cancer, and its inhibitors (such as GSK1363089 and crizotinib) can destroy tumor cells via a synergistic lethal pathway.^27^ A clinical trial (NCT04551495) investigating this is currently underway.^28^

Hou et al.^29^ studied the cBioPortal for Cancer Genomics and next‐generation sequencing (NGS) test results of 177 patients with lung adenocarcinoma and found the frequent occurrence of *ROS1* gene rearrangements in younger patients (<40 years old; *p* = 0.035); however, the significance of these mutations was unclear. Consistently, Wang et al.^30^, ^31^ also suggested that *ROS1* gene amplification or rearrangement mutations occurred more frequently in malignant tumors, such as gastrointestinal stromal tumors, gallbladder cancer, and soft tissue sarcoma. However, the significance and impact of these mutations require further investigation.

**FIGURE 2:**
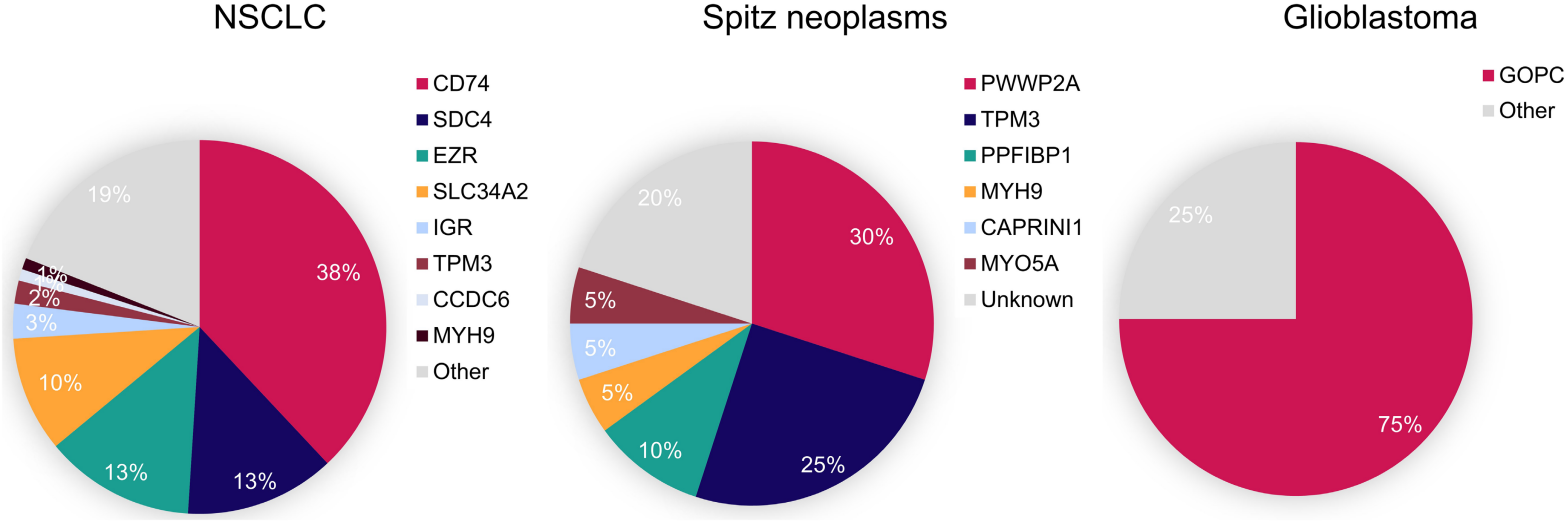
Pie chart of ROS1 fusion partner frequencies in non‐small cell lung cancer (NSCLC), Spitz neoplasms, and glioblastoma (percentages shown).

Compared to the overexpression and amplification of the *ROS1* gene, researchers believe that *ROS1* gene fusion is the primary driving force for tumorigenesis and disease progression.^4^, ^13^, ^32^, ^33^ Several *ROS1* fusion genes have been identified in NSCLC, Spitz neoplasms, and glioblastomas (Figure 2). In NSCLC, CD74 is the most common fusion partner of the *ROS1* gene, accounting for approximately 38% of cases, followed by EZR (13%), SDC4 (13%), and SLC34A2 (10%).^7^ Gerami et al.^34^ found that the PWWP2A‐ROS1 fusion had the highest frequency among 16 cases of Spitz neoplasm. In addition, GOPC‐ROS1 is the most common fusion in glioblastoma (75%)^35^; Richardson et al.^36^ suggested that this fusion may be caused by a microdeletion in chromosome 6q22.1. In conclusion, the high‐frequency ROS1 fusion partners differ among cancer types and are related to the intrinsic levels of fusion gene activation and fusion sites. For example, unlike in glioblastomas, *ROS1* gene fusion in NSCLC happens via interchromosomal translocations.^37^, ^38^ Differences in fusion partners lead to changes in subcellular localization, which affect downstream signaling pathways. For example, compared to CD74‐ROS1, which acts on the endoplasmic reticulum, SDC4‐ROS1 and SLC34A2‐ROS1 have a stronger ability to activate the MAPK pathway.^39^ Furthermore, there exists a mutually exclusive relationship between ROS1 fusions and other oncogenic mutations, a trend observed in NSCLC,^40^ gliomas,^41^ and Spitz neoplasms.^42^ ROS1 is rarely co‐mutated with the *ALK*, *EGFR*, or *KRAS* genes.^40^ Despite the similarity and high homology of the ROS1 and ALK domains, ROS1/ALK double fusions rarely coexist.^43^

## 3 DIAGNOSIS OF *ROS1* GENE MUTATIONS

*ROS1* gene mutations can occur in a variety of tumors, including NSCLC (1%–2%),^44^ gliomas (6%–7%),^45^ and cholangiocarcinomas (1.1%),^46^ among which ROS1 fusion NSCLC is the most common. A meta‐analysis of 9898 patients with NSCLC indicated that ROS1 fusion occurred in younger female patients with no smoking history and was more prevalent in patients with advanced disease (stage III–IV) than those with early disease (*p* < 0.001).^47^ Therefore, the prompt and effective diagnosis of ROS1 mutations is crucial. Several techniques have been reported for detecting ROS1 mutations that can be used for clinical screening or definitive diagnosis, as discussed in the succeeding sections.

### 3.1 Immunohistochemical staining

Immunohistochemical staining (IHC) is a useful screening tool for ROS1 fusions. ROS1 IHC can be performed using cellular specimens, paraffin‐embedded tissue specimens, and cell blocks for lung cancer detection.^48^ The most commonly used antibody for detecting ROS1 is the D4D6 rabbit monoclonal antibody (Cell Signaling Technology, Danvers, MA, USA), which has a sensitivity and specificity of 89% and 98%, respectively.^49^ A multicenter evaluation by Conde et al.^50^ found the SP384 antibody (Ventana, Tuscon, Arizona, USA) to be more sensitive (93%) in detecting ROS1 gene rearrangements than the D4D6 antibody. However, Hofman's experimental results suggested that D4D6 is more accurate for ROS1 rearrangements.^51^ Therefore, the use of these antibodies in IHC remains controversial. IHC results are mainly reported in the literature as 1+ (weak cytoplasmic staining), 2+ (moderate staining), or 3+ (strong staining) with positive microscopic manifestations of granular cytoplasmic staining.^52^ IHC is an inexpensive and highly accurate screening method; however, it lacks clear scoring criteria and can be expressed at different levels in different tumor cells. In addition, normal tissues, such as type II alveolar and osteoclast‐type giant cells, can also weakly express ROS1, which interferes with diagnosis.^53^

### 3.2 Reverse transcription‐polymerase chain reaction

Reverse transcription‐polymerase chain reaction (RT‐PCR) detects ROS1 rearrangements by identifying fusion mRNA and distinguishing fusion partners.^54^ RT‐PCR can be used to extract RNA from formalin‐fixed paraffin‐embedded tissue samples and cellular specimens,^55^ requires fewer tissue samples, and can be easily performed within a shorter detection period. However, RT‐PCR can only detect known fusion partners; it cannot identify unknown species of fusion genes.^56^ Owing to the large number of unknown ROS1 fusion partners,^57^ use of RT‐PCR has certain limitations in clinical applications.

### 3.3 Fluorescence in situ hybridization

Fluorescence in situ hybridization (FISH) is currently the only technique that can detect almost all ROS1 mutations.^58^ It involves using two probes labeled with different fluorescent colors (usually red, green, or orange) to target the 5′ and 3′ ends of ROS1.^59^ ROS1 fusion manifests in FISH as a loss of the probe at the 5′ end or a break in the signal, separating the two colors.^60^ An advantage of FISH is its ability to detect unknown fusion partners of ROS1. Furthermore, the cycle time, specificity, and sensitivity of FISH are clinically satisfactory.^59^ However, FISH is expensive, and the requirements for tissue specimens are stringent, such as tissue sections from infants under 6 months of age.^56^ Furthermore, FISH must be detected in more than 50 cell nuclei for diagnostic significance.^61^ In addition, some fusion partners of ROS1 (GOPC‐ROS1) cannot be detected by FISH owing to the limitations in the probe design^62^; self‐designed probes can overcome this limitation.^63^, ^64^ However, FISH remains the gold standard for detecting *ROS1* gene mutations.

### 3.4 Next‐generation sequencing

The range of NGS technologies, from analysis of mutant gene regions to whole‐genome sequencing, and their ability to detect multiple variant forms in parallel^65^ save time and tissue samples compared to single‐target detection. NGS can detect single nucleotide mutations, gene insertions/deletions, and genomic rearrangements, and both DNA and RNA can be used as samples.^66^ NGS is currently used to detect *ROS1* gene rearrangements, particularly for ROS1 fusion genes with negative FISH results, such as GOPC‐ROS1.^67^ Additionally, NGS can be performed using cell‐free DNA (cfDNA) or circulating tumor DNA (ctDNA), which are valid tools for early diagnosis.^68^ However, limitations exist in employing NGS technology for large‐scale clinical applications. First, NGS is expensive when analyzing only a few genes.^69^ Second, DNA‐only detection is less accurate and does not cover intronic breakpoints with large amounts of repetitive nucleotides, which whole‐genome RNA sequencing can overcome.^70^ Benayed et al.^71^ used an RNA‐based genome sequencer to test samples from 2522 patients with lung adenocarcinoma and found an improvement in the detection rate of ROS1 rearrangements compared to that of DNA samples. Finally, no standardized NGS process exists for the different types and stages of tumor progression, so additional clinical information is needed to define rare variants.

In summary, each of these four detection methods has advantages and disadvantages (Table 1), and there is no definitive conclusion regarding which assay should be used to detect ROS1 mutations. Owing to the tendency of ROS1 to be mutually exclusive with genes such as *ALK*, this study suggests that IHC can be used to screen tumor specimens negative for *ALK* expression before conducting FISH on specimens with positive IHC results. By contrast, NGS may be more appropriate for cases requiring early diagnosis and guidance on drug use.

**TABLE 1:** Advantages and disadvantages of ROS1 mutation detection technology.

Diagnostic techniques	Advantages	Limitations	Quotes
IHC	High sensitivity and specificity Wide range of selected materials Low cost	Absence of definitive diagnostic criteria Some normal tissues are also positively expressed, interfering with diagnosis	[48–53]
RT‐PCR	Low tissue requirements. Short testing cycles and simplicity in operation.	Unable to detect unknown types of fusion partners	[54–57]
FISH	Known or unknown fusion partners can be detected Short testing period High sensitivity and specificity	High cost Stringent requirements for tissue specimens Undetectable partial fusion genes	[56,58–64]
NGS	Possibility of parallel testing, saving time and tissue samples Partial detection of FISH‐negative fusion types Detectable ctDNA for early diagnosis	High cost Requires combined RNA sequencing to complement the range of detection No standardized process	[65–71]

Abbreviations: ctDNA, circulating tumor DNA; FISH, fluorescence in situ hybridisation; IHC, immunohistochemical staining; NGS, next‐generation sequencing; RT‐PCR, reverse transcription polymerase chain reaction.

## 4 ROS1 KINASE INHIBITORS

All ROS1 inhibitors are multi‐kinase inhibitors that can inhibit ALK, MET, and other kinases (such as EGFR, JAK2, and TrkA) in addition to ROS.^72^ Early‐stage ROS1 TKIs, such as crizotinib, ceritinib, and entrectinib, can be used in patients with ROS1‐positive NSCLC who have not received TKI treatment (TKI‐naïve).^10^, ^73^ Considering resistance mutations are inevitable during treatment with early‐stage TKIs, next‐generation TKIs such as cabozantinib and taletrectinib have been tested in clinical trials. However, most of these are not approved for ROS1‐positive tumors.^74^, ^75^, ^76^

ROS1 kinase inhibitors primarily affect the kinase domain of ROS1.^77^ Conventional kinases can be classified into DFG‐in (active, type I) and DFG‐out (inactive, type II) kinases, depending on the domain conformation. DFG refers to the activation loop that regulates kinase activity, consisting of aspartic acid (D), phenylalanine (F), and glycine (G).^78^ When the domain is in the DFG‐in state, aspartate rotates inward to expose the ATP‐binding site. Most TKIs (crizotinib, ceritinib, entrectinib, taletrectinib, and repotrectinib) compete for the ATP‐binding site, preventing further phosphorylation of the kinase by ATP, thus inhibiting kinase activity.^79^ In the DFG‐out state, the aspartic acid in the domain is rotated outward and exposes a hydrophobic site that can be occupied by a few TKIs (such as cabozantinib and foretinib) to inhibit tumor activity.^79^ As the DFG‐out conformation does not need to participate in the catalytic reaction, resulting in less restricted structure and providing greater selectivity in the design of inhibitors. However, the development of type II kinase inhibitors remains challenging because most kinases lack the DFG‐out domain conformation.

Both type I and type II ROS1 kinase inhibitors have inhibitory effects on ROS1 fusion‐positive tumors,^80^ including crizotinib in CD74‐ROS1 and SDC4‐ROS1 fusion NSCLC,^81^ ceritinib in TFG‐ROS1 fusion inflammatory myofibroblastoma,^82^ and repotrectinib in G2032R‐ROS1 and D2033N‐ROS1 fusion tumors.^83^ Furthermore, studies by Davare and Facchinetti have shown slight differences in the inhibitory effects of TKIs on different ROS1 fusion phenotypes.^84^, ^85^ However, a statistical review of clinical trial data by Drilon et al.^13^ revealed that differences in the level of inhibition did not affect treatment efficacy.

## 5 TREATMENT OF ROS1‐MUTANT TUMORS

### 5.1 NSCLC

**FIGURE 3:**
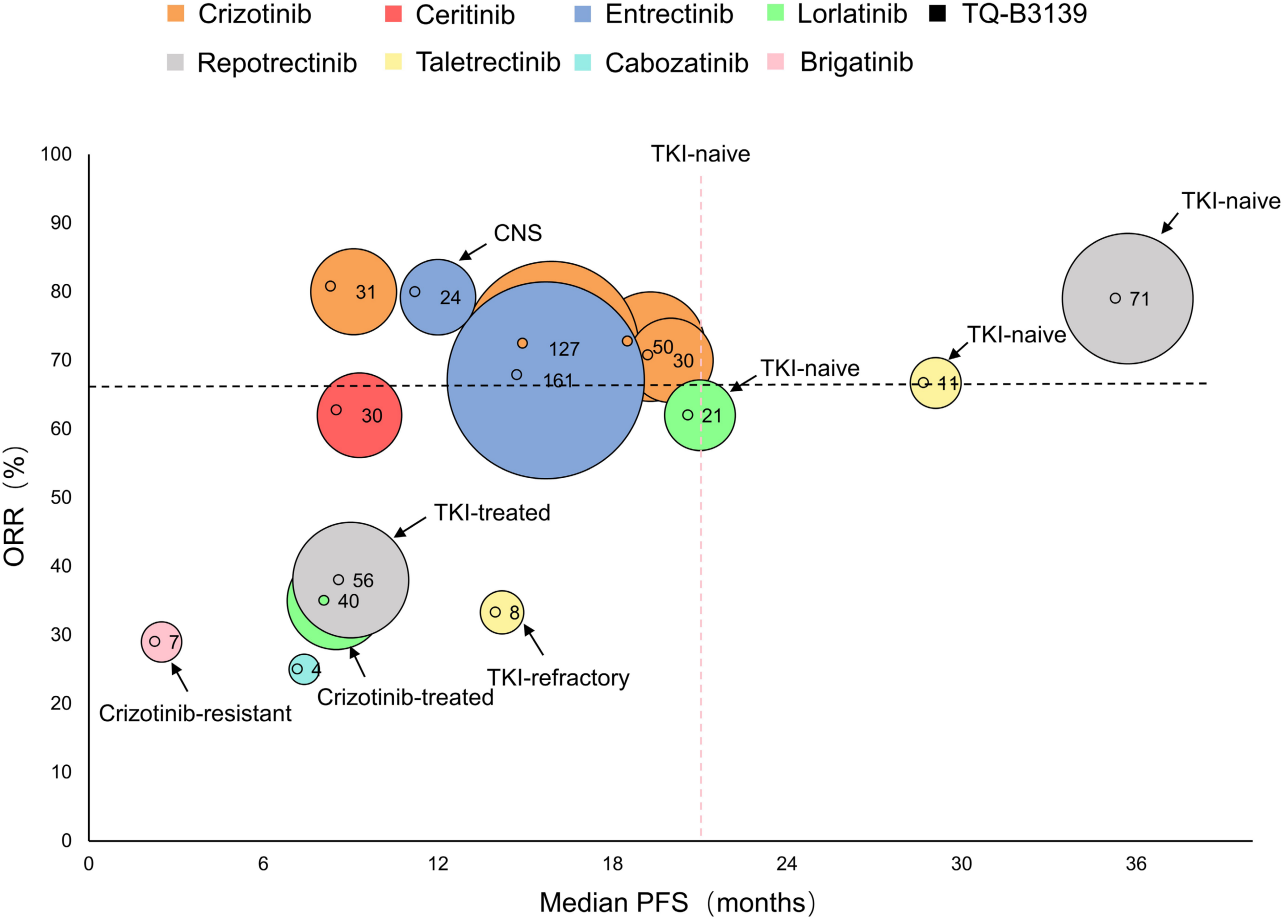
Activity of tyrosine kinase inhibitors (TKIs) in clinical trials involving ROS1 rearrangement‐positive non‐small cell lung cancer (NSCLC), comparing the objective remission rates (ORRs) and progression‐free survival (PFS). Unclear ORRs or PFS are indicated with dashed lines.

NSCLC accounts for approximately 80% of all lung cancers, and ROS1 fusion‐positive NSCLC accounts for 1%–2% of NSCLC cases.^86^ Currently, ROS1 kinase inhibitors are the first‐line treatment for advanced ROS1 fusion‐positive NSCLC^87^ (Table 2 and Figure 3).

**TABLE 2:** Summary of clinical trials for TKIs in ROS1 rearrangement‐positive NSCLC.

Drug	Clinical trial identifier/Study	Study design	ORR	Median DOR (months)	Median PFS (months)
Crizotinib	NCT00585195	Interventional, parallel‐cohort, Open‐label Phase I trial	72%	24.7	19.3
	NCT01945021	Interventional Single‐group assignment Open‐label Phase II Single‐arm trial	71.7%	19.7	15.9
	EUROS1	Single‐group assignment Open‐label	80%	–	9.1
	NCT02183870/EUCROSS	Interventional, Single‐group assignment Open‐label Multicentre Phase II trial	70%	–	20.0
Ceritinib	NCT01964157	Interventional Open‐label Multicentre Phase II trial	62%	21.0	9.3
Entrectinib	ALKA‐372‐001, STARTRK‐1, STARTRK‐2	Interventional Open‐label Multicentre Phase I/II trial	67.1% 79.2% (CNS)	15.7 12.9 (CNS)	15.7 12.0 (CNS)
Lorlatinib	NCT01970865	Interventional Non‐randomized Open‐label Multicentre Phase I/II Single‐arm trial	41%	25.3	21.0 (TKI‐naïve) 8.5 (crizotinib‐treated)
Repotrectinib	NCT03093116	Interventional Single‐group assignment Open‐label Multicentre Phase I/II trial	79% (TKI‐naïve) 38% (TKI‐treated)	34.1 (TKI‐naïve) 14.8 (TKI‐treated)	35.7 (TKI‐naïve) 9.0 (TKI‐treated)
Taletrectinib	NCT02279433, NCT02675491	Interventional Non‐randomized Single‐group assignment Open‐label Multicentre Phase I/IB trial	66.7% (TKI‐naïve) 33.3% (TKI‐refractory)	23.5 (TKI‐naïve) 14.0 (TKI‐refractory)	29.1 (TKI‐naïve) 14.2 (TKI‐refractory)
TQ‐B3139	NCT03099330	Interventional Single‐group assignment Phase I trial Open‐label	66.7%	–	20.2 (partial response) 27.0 (complete response)
Cabozantinib	Sun et al.	–	25%	–	7.4
Brigatinib	Dudnik et al.	–	29% (crizotinib‐resistant)	–	21.6 (TKI‐naïve) 2.5 (crizotinib‐resistant)

In the phase I PROFILE 1001 clinical trial, 50 patients with advanced NSCLC treated with standard doses of crizotinib had an objective remission rate (ORR) of 72%, a median duration of remission (DOR) of 17.6 months, and a mean progression‐free survival (PFS) of 19.2 months.^88^ Based on the significant antitumor activity observed in this trial, crizotinib was the first drug approved by the FDA for advanced ROS1 rearrangement‐positive NSCLC.^88^ Recent reports from this trial showed an increased DOR of 24.7 months and PFS of 19.3 months after crizotinib treatment, with similar adverse effects, including visual impairment, edema, vomiting, and diarrhea.^89^ A phase II single‐arm trial (NCT01945021) of 127 patients with ROS1 rearrangement‐positive NSCLC in East Asia by Wu et al.^57^ reported an ORR of 71.7% and a mean PFS of 15.9 months for crizotinib. Another result from the EUROS1 cohort demonstrated an ORR of 80% and a mean PFS of 9.1 months in patients treated with crizotinib.^90^ In the follow‐up EUCROSS cohort phase II study (NCT02183870), the ORR was 70% in 30 patients, and PFS improved to 20.0 months.^91^ This suggests that crizotinib is highly effective in NSCLC patients of different ethnicities. However, most patients with advanced NSCLC treated with crizotinib eventually experience disease progression owing to ROS1 resistance mutations or CNS metastases.

Ceritinib is currently approved for patients with ALK rearrangement‐positive NSCLC resistant to crizotinib^92^, ^93^; however, it is also effective in patients with ROS1 rearrangement‐positive NSCLC. A phase II trial (NCT01964157) comprising 30 patients with advanced ROS1 rearrangement‐positive NSCLC not treated with crizotinib indicated that after a standard dose of ceritinib treatment (750 mg once daily), patients exhibited an ORR of 62%, a DOR of 21 months, and a mean PFS of 9.3 months; the common adverse events reported were gastrointestinal symptoms (78% diarrhea, 59% nausea, and 56% anorexia).^94^ As a selective ROS1 inhibitor, ceritinib is 20 times more effective than crizotinib.^95^ However, despite promising trial data, ceritinib may not work against crizotinib‐resistant mutations, including G2032R, D2033N, and L2086F.^80^, ^96^ In addition, ceritinib has a higher incidence of adverse effects than crizotinib.^94^, ^97^, ^98^

Entrectinib is a multi‐kinase inhibitor that inhibits ROS1, ALK, and TRK.^99^ According to an analysis of three phase I and II clinical trials (ALKA‐372‐001, STARTRK‐1, and STARTRK‐2), entrectinib had an ORR of 67.1% and a DOR and PFS of 15.7 months. For those who developed CNS metastases, the ORR was 79.2%, DOR was 12.9 months, and PFS was 12.0 months, with primary adverse effects including weight gain and neutropenia.^100^, ^101^ Unlike crizotinib, entrectinib has a weaker interaction with P‐glycoprotein (P‐gp) and can thus cross the blood–brain barrier, yielding ideal blood concentrations in the CNS and efficacy in patients with NSCLC who develop intracranial metastases.^102^, ^103^ Based on the positive clinical trial results and the specificity of the drug itself, entrectinib has become the second targeted agent approved by the FDA for treating patients with advanced ROS1 rearrangement‐positive NSCLC.^104^ However, similar to ceritinib, entrectinib appears to be ineffective in crizotinib‐resistant cases, particularly in the absence of activity in G2032R‐ and L2026M‐mutant tumor cells.^73^, ^105^

Other next‐generation TKIs also play an indispensable role in the treatment of ROS1 rearrangement‐positive NSCLC, including lorlatinib, repotrectinib, taletrectinib, TQ‐B3139, cabozantinib, and brigatinib. The ORR of lorlatinib in a single‐arm phase I‐II trials (NCT01970865) was 62% for TKI‐naïve cases and 35% for crizotinib‐treated cases, PFS was 21.0 months for TKI‐naïve cases compared to 8.5 months for crizotinib‐treated cases; the common adverse effects include hypertriglyceridemia (19%) and hypercholesterolemia (14%).^106^ A phase I–II trial of repotrectinib (NCT03093116) showed an ORR of 79%, PFS of 35.7 months, and DOR of 34.1 months in TKI‐naïve cases. Repotrectinib was also shown to be effective in patients who had previously received ROS1 TKI and had never received chemotherapy, with ORR of 38%, PFS of 9.0 months, and DOR of 14.8 months. The main adverse reactions were dizziness (58%), dysgeusia (50%), and paresthesia (30%).^107^ Phase I studies of taletrectinib in the United States (NCT02279433) and Japan (NCT02675491) showed an ORR of 66.7% and a mean PFS of 29.1 months in patients with TKI‐primary ROS1‐mutated NSCLC compared with an ORR of 33.3% and a PFS of 14.2 months in crizotinib‐resistant cases; its adverse effects included abnormal liver function (72.7%) and gastrointestinal symptoms (50%).^108^ The clinical activity of TQ‐B3139 as a novel TKI was validated. A phase I trial involving four patients with ROS1 fusion NSCLC (NCT03099330) showed that TQ‐B3139 had an ORR of 66.7%, along with a PFS of 20.2 months (partial response) or 27.0 months (complete response).^109^ The results of trials with cabozantinib (ORR, 25%; PFS: 4.9–13.8 months)^110^ and brigatinib (ORR, 29%)^111^ have also demonstrated potent antitumor activity. Next‐generation TKIs also performed well in crizotinib‐resistant NSCLC, such as repotrectinib for the G2032R/D2033N fusion mutation,^83^ lorlatinib and taletrectinib for the G2032R fusion mutation,^112^, ^113^ and cabozantinib and brigatinib for the CD74 fusion mutation.^114^, ^115^ Despite these promising results, only repotrectinib has been approved by the FDA as a first‐line agent for ROS1 rearrangement‐positive NSCLC.^103^ Further trials are necessary to demonstrate the efficacy and safety of next‐generation TKIs for clinical use.

The role of immunotherapy in ROS1‐positive NSCLC is not well‐defined. Choudhury et al. found that ROS1 regulates the expression of programmed cell death 1 ligand 1 (PD‐L1) by activating the MEK‐ERK and ROS1‐SHP2 pathways. However, most ROS1‐positive NSCLC cells do not express PD‐L1 and have a low mutation load.^116^ Immunotherapy combined with chemotherapy elicited a higher ORR in patients with ROS1‐positive NSCLC compared with immunotherapy alone.^117^, ^118^ In addition, a phase II clinical trial of Atezolizumab (NCT04042558) showed that Atezolizumab with or without bevacizumab and the “platinum‐Pemetrexed” chemotherapy regimen are effective in NSCLC patients with metastatic EGFR mutation or ALK/ROS1 rearrangement after TKI failure.^119^ This suggests that immunotherapy combined with chemotherapy is a potential treatment strategy for patients with ROS1‐positive NSCLC, especially those for whom TKI therapy has failed.

### 5.2 Non‐NSCLC tumors

ROS1 kinase inhibitors are also effective in non‐NSCLC tumors; however, owing to the low incidence of ROS1 rearrangements, no relevant TKIs are currently approved for treating non‐NSCLC tumors. This has also been mentioned in the literature as a case report (Table 3). For example, crizotinib has shown good or partial remission in YWHAE1‐ROS1 fusion inflammatory myofibroblastoma (IMT),^120^ GOPC‐ROS1 fusion Spitz naevi, high‐grade serous ovarian cancer,^67^, ^121^ RDX‐ROS1 fusion intrahepatic cholangiocarcinoma,^122^ and TJP1‐ROS1 fusion malignant peripheral nerve sheath tumors(MPNST).^123^ Cases on the use of entrectinib for ER+/HER2‐breast cancer, ARCN1‐ROS1 and ZCCHC8‐ROS1 fusion pediatric glioma, GOPC‐ROS1 fusion limbal melanoma, and SLC4A4‐ROS1 fusion metastatic pancreatic cancer have also been reported.^28^, ^124^, ^125^, ^126^, ^127^ Among the next‐generation TKIs, lorlatinib has been suggested to be potentially effective in ROS1 p.L1950F point‐mutated pancreatic cancer and TFG‐ROS1 fusion IMT of the chest wall,^128^, ^129^ with no relevant case reports for other TKIs.

**TABLE 3:** Overview of ROS1 kinase inhibitor therapy in non‐NSCLC tumors.

Drug	Study	Tumor histology	Fusion/Mutation	Response
Crizotinib	Comandini et al.	IMT	YWHAE1‐ROS1	PR
	Robertson et al.	Spitz nevi	GOPC‐ROS1	PR
	Dong et al.	High‐grade serous ovarian cancer	GOPC‐ROS1	PR
	Jakubowski et al.	Intrahepatic cholangiocarcinoma	RDX‐ROS1	PR
	Li et al.	MPNST	TJP1‐ROS1	SD
Entrectinib	Agostinetto et al.	ER+/HER2‐breast cancer (invasive lobular carcinoma)	E‐cadherin defect	PR
	Mayr et al.	Pediatric high‐grade glioma	ARCN1‐ROS1	PR
	Papusha et al.	Infant hemispheric glioma	ZCCHC8‐ROS1	PR
	Couts et al.	Acral lentiginous melanoma	GOPC‐ROS1	PR
	Pishvaian et al.	Metastatic pancreatic cancer	SLC4A4‐ROS1	SD
Lorlatinib	Velthaus et al.	Pancreatic cancer	ROS1 p.L1950F mutation	SD
	Carcamo et al.	IMT of the chest wall	TFG‐ROS1	PR

Abbreviations: IMT, inflammatory myofibroblastoma; MPNST, malignant peripheral nerve sheath tumor; PR, partial response; SD, stable disease.

## 6 CHALLENGES OF ROS1‐TARGETED THERAPY

### 6.1 Adverse effects related to ROS1 inhibitors

Adverse events (AEs) are a major challenge in ROS1‐targeted therapy. The use of most ROS1 kinase inhibitors in clinical practice has been limited because of the AEs associated with their mechanism of action (Table 4), such as edema, visual disorders, gastrointestinal symptoms, and, in severe cases, liver function abnormalities, neutropenia, and even life‐threatening effects.^130^, ^131^ Some AEs are related to the unique systems in which TKIs act. For example, entrectinib inhibits TRK and acts on the nervous system, resulting in dizziness, weight gain, and cognitive impairment during targeted therapy.^132^ Moreover, crizotinib can cause peripheral edema in patients because of MET kinase inhibition.^133^, ^134^ However, other adverse reactions still lack clear mechanisms to explain their occurrences, such as abnormal liver function,^135^ manifestations of ocular toxicity associated with crizotinib treatment,^136^ hyperlipidemia, and peripheral neuropathy associated with lorlatinib treatment.^131^ There is currently no standardized clinical treatment process to mitigate the damage caused by AEs. Bauer et al.^131^ suggested that adjusting drug doses or symptomatic supportive treatment could manage mild to moderate AEs (grade <3). Although next‐generation TKIs are considered to have potential value in reducing the incidence of AEs owing to their high affinity,^13^ recent studies have demonstrated that next‐generation TKIs do not have an absolute advantage in treating AEs.^137^, ^138^

**TABLE 4:** Summary of AEs associated with clinical trials of ROS1 inhibitors.

Drug	Study	Overall AEs in ≥20% of patients	Treatment‐related AEs (≥ grade 3)
Crizotinib	PROFILE 1001	Vision disorder (87%) Nausea (51%) Oedema (47%) Diarrhea (45%) Vomiting (38%) Elevated transaminases (36%) Constipation (34%) Bradycardia (21%) Fatigue (21%)	Hypophosphatemia (15%) Neutropenia (9%) Vomiting (4%) Elevated transaminases (4%)
	NCT01945021	Elevated transaminases (55.1%) Vision disorder (48%) Nausea (40.9%) Diarrhea (38.6%) Vomiting (32.3%) Constipation (29.9%) Neutropenia (29.1%) Leukopenia (22.8%) Oedema (22.8%)	Neutropenia (10.2%) Elevated transaminases (5.5%) Leukopenia (2.4%) Nausea (1.6%)
	EUCROSS	Vision disorder (65%) Diarrhea (56%) Oedema (50%) Bradycardia (47%) Nausea (41%) Increased ALT (35%) Vomiting (32%) Leukopenia/neutropenia (32%) Increased AST (26%) Increased blood creatinine (21%)	Leukopenia/neutropenia (9%) Nausea (3%) Vomiting (3%) ALT increased (3%) Dysgeusia (3%) Pulmonary embolism (3%)
Ceritinib	NCT01964157	Diarrhea (78%) Nausea (62%) Anorexia (59%) Vomiting (53%) Cough (47%) Pain (41%) Fatigue (38%) Dyspnoea (25%)	Fatigue (16%) Pneumonia (12%) Dry mouth (3%) Pleural effusion (3%)
Entrectinib	NCT02097810, NCT02568267	Dysgeusia (43.4%) Dizziness (34.8%) Constipation (31.4%) Fatigue (30%) Weight gain (28.6%) Diarrhea (26.7%)	Weight gain (8.1%) Increased ALT (3.3%) Diarrhea (2.9%) Increased AST (2.4%) Decreased neutrophil count (2.4%) Neutropenia (1.9%) Rash (1.4%)
Lorlatinib	NCT01970865	Hypercholesterolaemia (72%) Hypertriglyceridemia (39%) Peripheral oedema (39%) Peripheral neuropathy (39%) Cognitive effects (22%)	Hypercholesterolaemia (13%) Hypertriglyceridemia (6%) Weight gain (6%) Increased lipase (4%) Cognitive effects (2%) Increased AST (2%)
Repotrectinib	NCT03093116	Dizziness (58%) Dysgeusia (50%) Paresthesia (30%) Constipation (26%) Anemia (26%) Ataxia (20%)	Anemia (4%) Increased blood creatine kinase leve (4%) Dizziness (3%) Weight increase (2%)
Taletrectinib	NCT02279433, NCT02675491	Increased ALT (72.7%) Increased AST (72.7%) Nausea (59.1%) Diarrhea (54.5%) Vomiting (36.4%) Increased blood creatinine (31.8%)	Increased ALT (22.7%) Increased AST (13.6%) Diarrhea (4.5%)

### 6.2 Resistance to ROS1 inhibitors

The prolonged use of ROS1 inhibitors often leads to the development of drug resistance in tumor cells during disease progression. The mechanisms leading to the development of drug resistance in tumors mainly include mutations in the kinase domain and activation of collateral signaling.^139^, ^140^ Point mutations in the structural domain of the ROS1 kinase can transform the drug target and reduce its inhibitory effect, leading to resistance to targeted therapies.^141^ G2032R was the first identified and most common drug resistance mutation in ROS1.^142^ The mutation results in the substitution of glycine with an arginine that still enables ATP binding but conflicts with the piperidine ring structure of crizotinib, causing resistance to ROS1 kinase inhibition, mediating epithelial‐mesenchymal transition, and upregulating Twist 1, contributing to tumor progression.^141^, ^143^, ^144^ Another mutation, D2033N, causes drug resistance by affecting the solvent front region of the ATP‐binding site.^145^ In addition, other drug resistance mutations, such as L2155S, affect protein function to enhance drug resistance,^143^ and S1986F/Y (parallel mutation) and L2026M, promote kinase activity by blocking the critical binding site.^85^, ^146^ This mechanism of action accounts for 50%–60% of ROS1‐resistant tumors.

**FIGURE 4:**
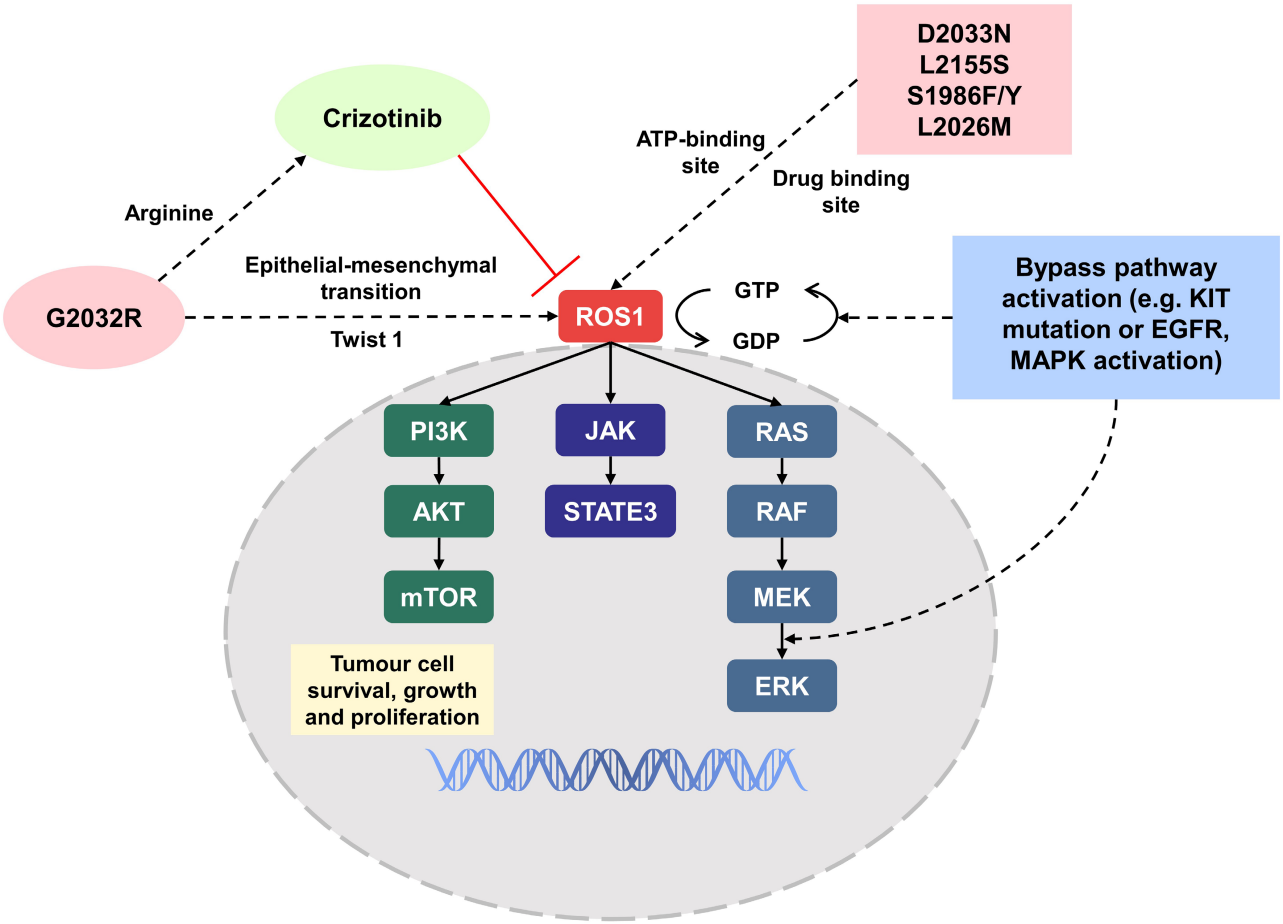
Molecular mechanisms of resistance to ROS1 tyrosine kinase inhibitors (TKIs), depicting the two main drug resistance patterns.

The activation of bypass signaling pathways (e.g., the EGFR pathway) has been shown to confer drug resistance by reducing tumor dependence on ROS1 activity and increasing its dependence on the self.^147^, ^148^ Including previously reported mutations in the KIT and MAPK pathways, autophosphorylation or alterations in the MEK/ERK pathway can render tumor cells resistant to crizotinib^8^, ^149^ (Figure 4).

In addition, resistance to ROS1 inhibitors can lead to the phenotypic transformation of tumor cells.^150^ Lin et al. reported a case of a patient with NSCLC whose pathological classification transformed into adenocarcinoma after ROS1 inhibitor resistance; this phenotypic change may be related to retinoblastoma‐1 (RB1) and TP53 inactivation.^151^

The current mainstream response in response to kinase inhibitor resistance is to replace next‐generation TKIs or combine them with other kinase inhibitors. Yun et al.^76^ found that repotrectinib has a targeted therapeutic effect against the G2032R mutation with a similar response observed with foretinib.^84^ Lorlatinib has therapeutic effects against other mutations, such as L2026M and S1986F/Y.^152^ In particular, combinations of kinase inhibitors appeared to be more effective against resistance due to the activation of bypass signaling pathways. Dziadziuszko et al.^149^ discovered that combining crizotinib with ponatinib could overcome resistance caused by mutations in the KIT pathway in vitro. Vaishnavi et al.^140^ detected the expression of the EGFR pathway in SLC34A2‐ROS1 fusion HCC78 cells, and the coadministration of crizotinib with the EGFR inhibitor gefitinib reduced drug resistance in HCC78 cells.^153^ Aside from point mutations in the structural domain of ROS1 and the activation of bypass signaling pathways, some unknown drug resistance mechanisms exist, such as lorlatinib resistance in patients with ROS1 fusion‐positive NSCLC.^80^ Therefore, when developing next‐generation TKIs, future studies should focus on epigenetics, RNA, and proteins to elucidate the mechanisms of ROS1 resistance.

### 6.3 Central nervous system metastases

CNS metastases are common in ROS1‐mutant malignancies. CNS metastases occur in approximately 36% of patients with stage IV ROS1‐positive NSCLC.^154^ Li et al.^155^ found a higher incidence of CNS metastasis in patients with NSCLC having CD74‐ROS1 fusion compared to non‐CD74‐ROS1 fusion cases (*p* = 0.020), suggesting that the type of fusion partner is associated with CNS metastasis. Jun et al.^156^ discovered that CD74‐ROS1 confers a higher metastatic potential to tumor cells by activating the phosphorylation of the plasma membrane protein E‐Syt1. Due to its poor ability to cross the blood–brain barrier, crizotinib cannot be used as the first drug of choice for CNS metastasis.^157^ However, next‐generation TKIs have exhibited great potential in cases of CNS metastases, including ceritinib, which showed an ORR of 25% in patients with CNS metastases in a phase II study,^94^ and lorlatinib, which showed an ORR of 50%.^106^ Among these, entrectinib is the most effective, with an ORR of 55% and a mean PFS of 13.6 months.^100^ In contrast to TKI replacement, CNS metastases are sensitive to radiotherapy. Hence, they can be an option for controlling the progression of CNS metastases when patients cannot tolerate targeted therapy or have few lesions.^73^

## 7 PROSPECTS AND CONCLUSIONS

As a proto‐oncogene, ROS1 is mostly expressed in malignant tumors such as NSCLC. Chromosomal rearrangements resulting in ROS1 fusions drive tumor progression, and an appropriate diagnosis should be made on a clinical basis. Only a few ROS1 kinase inhibitors are currently approved for use in NSCLC; however, the efficacy of other TKIs for NSCLC and other malignancies has not been ascertained, as they are still being tested in clinical trials. Next‐generation TKIs appear capable of overcoming resistance and delaying CNS metastasis owing to their high affinity; however, they are associated with a greater incidence of adverse effects. Further research on next‐generation TKIs regarding the localization of ROS1 and its fusion partners, binding sites for targeted drugs (type I vs. type II), and coadministration with other drugs is required. Moreover, the correlation between TKIs and chemotherapy or immunotherapy in clinical practice requires further study.

## AUTHOR CONTRIBUTIONS

**Shizhe Li:** Writing – original draft (equal). **He Zhang:** Methodology (equal); software (equal). **Ting Chen:** Data curation (equal); supervision (equal). **Xiaowen Zhang:** Writing – review and editing (equal). **Guanning Shang:** Writing – review and editing (equal).

## FUNDING INFORMATION

Shenyang Science and Technology Project (21–173–9‐24). China Postdoctoral Science Foundation (2021 M693912). 345 Talent Project of Shengjing Hospital (M0944). 345 Talent Project of Shengjing Hospital (M0744).

## CONFLICT OF INTEREST STATEMENT

The authors declare that they have no conflict of interests.

## Data Availability

The materials that support the conclusion of this review have been included within the article.

## References

[1] Birchmeier C , Sharma S , Wigler M . Expression and rearrangement of the ROS1 gene in human glioblastoma cells. Proc Natl Acad Sci USA. 1987;84(24):9270‐9274. 2827175 10.1073/pnas.84.24.9270 PMC299735

[2] Hubbard‐Smith K , Patsalis P , Pardinas JR , Jha KK , Henderson AS , Ozer HL . Altered chromosome 6 in immortal human fibroblasts. Mol Cell Biol. 1992;12(5):2273‐2281. 1373811 10.1128/mcb.12.5.2273-2281.1992 PMC364399

[3] Azelby CM , Sakamoto MR , Bowles DW . ROS1 targeted therapies: current status. Curr Oncol Rep. 2021;23(8):94. 34125313 10.1007/s11912-021-01078-y

[4] Uguen A , De Braekeleer M . ROS1 fusions in cancer: a review. Future Oncol. 2016;12(16):1911‐1928. 27256160 10.2217/fon-2016-0050

[5] Rikova K , Guo A , Zeng Q , et al. Global survey of phosphotyrosine signaling identifies oncogenic kinases in lung cancer. Cell. 2007;131(6):1190‐1203. 18083107 10.1016/j.cell.2007.11.025

[6] Wu K , Liao X , Gong Y , et al. Circular RNA F‐circSR derived from SLC34A2‐ROS1 fusion gene promotes cell migration in non‐small cell lung cancer. Mol Cancer. 2019;18(1):98. 31118036 10.1186/s12943-019-1028-9 PMC6530145

[7] Cui M , Han Y , Li P , et al. Molecular and clinicopathological characteristics of ROS1‐rearranged non‐small‐cell lung cancers identified by next‐generation sequencing. Mol Oncol. 2020;14(11):2787‐2795. 32871626 10.1002/1878-0261.12789 PMC7607175

[8] Sato H , Schoenfeld AJ , Siau E , et al. MAPK pathway alterations correlate with poor survival and drive resistance to therapy in patients with lung cancers driven by ROS1 fusions. Clin Cancer Res. 2020;26(12):2932‐2945. 32122926 10.1158/1078-0432.CCR-19-3321 PMC8034819

[9] Skoulidis F , Heymach JV . Co‐occurring genomic alterations in non‐small‐cell lung cancer biology and therapy. Nat Rev Cancer. 2019;19(9):495‐509. 31406302 10.1038/s41568-019-0179-8 PMC7043073

[10] D'Angelo A , Sobhani N , Chapman R , et al. Focus on ROS1‐positive non‐small cell lung cancer (NSCLC): Crizotinib, resistance mechanisms and the newer generation of targeted therapies. Cancers (Basel). 2020;12(11):3293. 33172113 10.3390/cancers12113293 PMC7694780

[11] Raedler LA . Zykadia (Ceritinib) approved for patients with Crizotinib‐resistant ALK ‐positive non‐small‐cell lung cancer. Am Health Drug Benefits. 2015;8(Spec Feature):163‐166. 26629283 PMC4665050

[12] Remon J , Pignataro D , Novello S , Passiglia F . Current treatment and future challenges in ROS1‐ and ALK‐rearranged advanced non‐small cell lung cancer. Cancer Treat Rev. 2021;95:102178. 33743408 10.1016/j.ctrv.2021.102178

[13] Drilon A , Jenkins C , Iyer S , Schoenfeld A , Keddy C , Davare MA . ROS1‐dependent cancers—biology, diagnostics and therapeutics. Nat Rev Clin Oncol. 2021;18(1):35‐55. 32760015 10.1038/s41571-020-0408-9 PMC8830365

[14] Springer TA . An extracellular beta‐propeller module predicted in lipoprotein and scavenger receptors, tyrosine kinases, epidermal growth factor precursor, and extracellular matrix components. J Mol Biol. 1998;283(4):837‐862. 9790844 10.1006/jmbi.1998.2115

[15] Shaw AT , Hsu PP , Awad MM , Engelman JA . Tyrosine kinase gene rearrangements in epithelial malignancies. Nat Rev Cancer. 2013;13(11):772‐787. 24132104 10.1038/nrc3612 PMC3902129

[16] Tan AC , Tan DSW . Targeted therapies for lung cancer patients with oncogenic driver molecular alterations. J Clin Oncol. 2022;40(6):611‐625. 34985916 10.1200/JCO.21.01626

[17] Wang W , He B . MiR‐760 inhibits the progression of non‐small cell lung cancer through blocking ROS1/Ras/Raf/MEK/ERK pathway. Biosci Rep. 2020;40:BSR20182483. 10.1042/BSR20182483 32347292

[18] Zong CS , Chan JL , Yang SK , et al. Mutations of Ros differentially effecting signal transduction pathways leading to cell growth versus transformation. J Biol Chem. 1997;272(3):1500‐1506. 8999820 10.1074/jbc.272.3.1500

[19] Acquaviva J , Wong R , Charest A . The multifaceted roles of the receptor tyrosine kinase ROS in development and cancer. Biochim Biophys Acta. 2009;1795(1):37‐52. 18778756 10.1016/j.bbcan.2008.07.006

[20] Keilhack H , Muller M , Bohmer SA , et al. Negative regulation of Ros receptor tyrosine kinase signaling. An epithelial function of the SH2 domain protein tyrosine phosphatase SHP‐1. J Cell Biol. 2001;152(2):325‐334. 11266449 10.1083/jcb.152.2.325 PMC2199605

[21] Charest A , Wilker EW , McLaughlin ME , et al. ROS fusion tyrosine kinase activates a SH2 domain‐containing phosphatase‐2/phosphatidylinositol 3‐kinase/mammalian target of rapamycin signaling axis to form glioblastoma in mice. Cancer Res. 2006;66(15):7473‐7481. 16885344 10.1158/0008-5472.CAN-06-1193

[22] Maxwell M , Galanopoulos T , Nevillegolden J , et al. Overexpression of the ros1 gene in primary human gliomas may contribute to malignant progression. Int J Oncol. 1996;8(4):713‐718. 21544418 10.3892/ijo.8.4.713

[23] Zhao JF , Sharma S . Expression of the ROS1 oncogene for tyrosine receptor kinase in adult human meningiomas. Cancer Genet Cytogenet. 1995;83(2):148‐154. 7553586 10.1016/0165-4608(95)00043-o

[24] Cheng Y , Sun Y , Wang LZ , et al. Cytoplasmic c‐ros oncogene 1 receptor tyrosine kinase expression may be associated with the development of human oral squamous cell carcinoma. Oncol Lett. 2015;10(2):934‐940. 26622599 10.3892/ol.2015.3340 PMC4509419

[25] Chang YJ , Chen KW , Chen L . Mitochondrial ROS1 increases mitochondrial fission and respiration in Oral squamous cancer carcinoma. Cancers (Basel). 2020;12(10):2845. 33019722 10.3390/cancers12102845 PMC7599653

[26] Bayard Q , Caruso S , Couchy G , et al. Recurrent chromosomal rearrangements of ROS1, FRK and IL6 activating JAK/STAT pathway in inflammatory hepatocellular adenomas. Gut. 2020;69(9):1667‐1676. 31907296 10.1136/gutjnl-2019-319790

[27] Bajrami I , Marlow R , van de Ven M , et al. E‐cadherin/ROS1 inhibitor synthetic lethality in breast cancer. Cancer Discov. 2018;8(4):498‐515. 29610289 10.1158/2159-8290.CD-17-0603 PMC6296442

[28] Agostinetto E , Nader‐Marta G , Paesmans M , et al. ROSALINE: a phase II, neoadjuvant study targeting ROS1 in combination with endocrine therapy in invasive lobular carcinoma of the breast. Future Oncol. 2022;18(22):2383‐2392. 35695563 10.2217/fon-2022-0358

[29] Hou H , Zhang C , Qi X , et al. Distinctive targetable genotypes of younger patients with lung adenocarcinoma: a cBioPortal for cancer genomics data base analysis. Cancer Biol Ther. 2020;21(1):26‐33. 31594446 10.1080/15384047.2019.1665392 PMC7012066

[30] Wang S , Chen R , Tang Y , et al. Comprehensive genomic profiling of rare tumors: routes to targeted therapies. Front Oncol. 2020;10:536. 32373528 10.3389/fonc.2020.00536 PMC7186305

[31] Chen HW , Chen TW . Genomic‐guided precision therapy for soft tissue sarcoma. ESMO Open. 2020;5(2):e000626. 32132106 10.1136/esmoopen-2019-000626 PMC7059546

[32] Takeuchi K , Soda M , Togashi Y , et al. RET, ROS1 and ALK fusions in lung cancer. Nat Med. 2012;18(3):378‐381. 22327623 10.1038/nm.2658

[33] Kohno T , Nakaoku T , Tsuta K , et al. Beyond ALK‐RET, ROS1 and other oncogene fusions in lung cancer. Transl Lung Cancer Res. 2015;4(2):156‐164. 25870798 10.3978/j.issn.2218-6751.2014.11.11 PMC4384213

[34] Gerami P , Kim D , Compres EV , et al. Clinical, morphologic, and genomic findings in ROS1 fusion Spitz neoplasms. Mod Pathol. 2021;34(2):348‐357. 32862201 10.1038/s41379-020-00658-w PMC7855005

[35] Sievers P , Stichel D , Sill M , et al. GOPC:ROS1 and other ROS1 fusions represent a rare but recurrent drug target in a variety of glioma types. Acta Neuropathol. 2021;142(6):1065‐1069. 34536122 10.1007/s00401-021-02369-1 PMC8568855

[36] Richardson TE , Tang K , Vasudevaraja V , et al. GOPC‐ROS1 fusion due to microdeletion at 6q22 is an oncogenic driver in a subset of pediatric gliomas and glioneuronal tumors. J Neuropathol Exp Neurol. 2019;78(12):1089‐1099. 31626289 10.1093/jnen/nlz093

[37] Bergethon K , Shaw AT , Ou SH , et al. ROS1 rearrangements define a unique molecular class of lung cancers. J Clin Oncol. 2012;30(8):863‐870. 22215748 10.1200/JCO.2011.35.6345 PMC3295572

[38] Ou SI , Nagasaka M . A catalog of 5' fusion partners in ROS1‐positive NSCLC circa 2020. JTO Clin Res Rep. 2020;1(3):100048. 34589944 10.1016/j.jtocrr.2020.100048 PMC8474457

[39] Neel DS , Allegakoen DV , Olivas V , et al. Differential subcellular localization regulates oncogenic signaling by ROS1 kinase fusion proteins. Cancer Res. 2019;79(3):546‐556. 30538120 10.1158/0008-5472.CAN-18-1492 PMC6359944

[40] Lin JJ , Ritterhouse LL , Ali SM , et al. ROS1 fusions rarely overlap with other oncogenic drivers in non‐small cell lung cancer. J Thorac Oncol. 2017;12(5):872‐877. 28088512 10.1016/j.jtho.2017.01.004 PMC5403618

[41] Clarke M , Mackay A , Ismer B , et al. Infant high‐grade gliomas comprise multiple subgroups characterized by novel targetable gene fusions and favorable outcomes. Cancer Discov. 2020;10(7):942‐963. 32238360 10.1158/2159-8290.CD-19-1030 PMC8313225

[42] Donati M , Kastnerova L , Martinek P , et al. Spitz tumors with ROS1 fusions: a clinicopathological study of 6 cases, including FISH for chromosomal copy number alterations and mutation analysis using next‐generation sequencing. Am J Dermatopathol. 2020;42(2):92‐102. 31361613 10.1097/DAD.0000000000001499

[43] Song Z , Zheng Y , Wang X , Su H , Zhang Y , Song Y . ALK and ROS1 rearrangements, coexistence and treatment in epidermal growth factor receptor‐wild type lung adenocarcinoma: a multicenter study of 732 cases. J Thorac Dis. 2017;9(10):3919‐3926. 29268402 10.21037/jtd.2017.09.79 PMC5723822

[44] Alexander M , Kim SY , Cheng H . Update 2020: Management of non‐small cell lung cancer. Lung. 2020;198(6):897‐907. 33175991 10.1007/s00408-020-00407-5 PMC7656891

[45] Guerreiro Stucklin AS , Ryall S , Fukuoka K , et al. Alterations in ALK/ROS1/NTRK/MET drive a group of infantile hemispheric gliomas. Nat Commun. 2019;10(1):4343. 31554817 10.1038/s41467-019-12187-5 PMC6761184

[46] Lim SM , Yoo JE , Lim KH , et al. Rare incidence of ROS1 rearrangement in cholangiocarcinoma. Cancer Res Treat. 2017;49(1):185‐192. 27121721 10.4143/crt.2015.497 PMC5266400

[47] Zhu Q , Zhan P , Zhang X , et al. Clinicopathologic characteristics of patients with ROS1 fusion gene in non‐small cell lung cancer: a meta‐analysis. Transl Lung Cancer Res. 2015;4(3):300‐309. 26207220 10.3978/j.issn.2218-6751.2015.05.01 PMC4483477

[48] Wang W , Cheng G , Zhang G , Song Z . Evaluation of a new diagnostic immunohistochemistry approach for ROS1 rearrangement in non‐small cell lung cancer. Lung Cancer. 2020;146:224‐229. 32580101 10.1016/j.lungcan.2020.06.019

[49] Yoshida A , Tsuta K , Wakai S , et al. Immunohistochemical detection of ROS1 is useful for identifying ROS1 rearrangements in lung cancers. Mod Pathol. 2014;27(5):711‐720. 24186139 10.1038/modpathol.2013.192

[50] Conde E , Hernandez S , Martinez R , et al. Assessment of a new ROS1 immunohistochemistry clone (SP384) for the identification of ROS1 rearrangements in patients with non‐small cell lung carcinoma: the ROSING study. J Thorac Oncol. 2019;14(12):2120‐2132. 31349061 10.1016/j.jtho.2019.07.005

[51] Hofman V , Rouquette I , Long‐Mira E , et al. Multicenter evaluation of a novel ROS1 immunohistochemistry assay (SP384) for detection of ROS1 rearrangements in a large cohort of lung adenocarcinoma patients. J Thorac Oncol. 2019;14(7):1204‐1212. 30999109 10.1016/j.jtho.2019.03.024

[52] Boyle TA , Masago K , Ellison KE , Yatabe Y , Hirsch FR . ROS1 immunohistochemistry among major genotypes of non‐small‐cell lung cancer. Clin Lung Cancer. 2015;16(2):106‐111. 25467930 10.1016/j.cllc.2014.10.003 PMC4770803

[53] Pavlakis N , Cooper C , John T , et al. Australian consensus statement for best practice ROS1 testing in advanced non‐small cell lung cancer. Pathology. 2019;51(7):673‐680. 31668406 10.1016/j.pathol.2019.08.006

[54] Shan L , Lian F , Guo L , et al. Detection of ROS1 gene rearrangement in lung adenocarcinoma: comparison of IHC, FISH and real‐time RT‐PCR. PLoS One. 2015;10(3):e0120422. 25742289 10.1371/journal.pone.0120422 PMC4351102

[55] Zhang L , Wang Y , Zhao C , et al. High feasibility of cytological specimens for detection of ROS1 fusion by reverse transcriptase PCR in Chinese patients with advanced non‐small‐cell lung cancer. Onco Targets Ther. 2019;12:3305‐3311. 31118681 10.2147/OTT.S198827 PMC6501702

[56] Bubendorf L , Buttner R , Al‐Dayel F , et al. Testing for ROS1 in non‐small cell lung cancer: a review with recommendations. Virchows Arch. 2016;469(5):489‐503. 27535289 10.1007/s00428-016-2000-3 PMC5082594

[57] Wu YL , Yang JC , Kim DW , et al. Phase II study of Crizotinib in East Asian patients with ROS1‐positive advanced non‐small‐cell lung cancer. J Clin Oncol. 2018;36(14):1405‐1411. 29596029 10.1200/JCO.2017.75.5587

[58] Chrzanowska NM , Kowalewski J , Lewandowska MA . Use of fluorescence in situ hybridization (FISH) in diagnosis and tailored therapies in solid tumors. Molecules. 2020;25(8):1864. 32316657 10.3390/molecules25081864 PMC7221545

[59] Hieggelke L , Schultheis AM . Application of FISH in the diagnosis of lung cancer. Pathologe. 2020;41(6):582‐588. 32989488 10.1007/s00292-020-00831-7

[60] Xu Y , Chang H , Wu L , et al. High prevalence of ROS1 gene rearrangement detected by FISH in EGFR and ALK negative lung adenocarcinoma. Exp Mol Pathol. 2020;117:104548. 32979347 10.1016/j.yexmp.2020.104548

[61] Fielder T , Butler J , Tierney G , et al. ROS1 rearrangements in lung adenocarcinomas are defined by diffuse strong immunohistochemical expression of ROS1. Pathology. 2022;54(4):399‐403. 34702583 10.1016/j.pathol.2021.07.012

[62] Rimkunas VM , Crosby KE , Li D , et al. Analysis of receptor tyrosine kinase ROS1‐positive tumors in non‐small cell lung cancer: identification of a FIG‐ROS1 fusion. Clin Cancer Res. 2012;18(16):4449‐4457. 22661537 10.1158/1078-0432.CCR-11-3351

[63] Antonescu CR , Suurmeijer AJ , Zhang L , et al. Molecular characterization of inflammatory myofibroblastic tumors with frequent ALK and ROS1 gene fusions and rare novel RET rearrangement. Am J Surg Pathol. 2015;39(7):957‐967. 25723109 10.1097/PAS.0000000000000404 PMC4465992

[64] Pisapia P , Lozano MD , Vigliar E , et al. ALK and ROS1 testing on lung cancer cytologic samples: perspectives. Cancer Cytopathol. 2017;125(11):817‐830. 28743163 10.1002/cncy.21899

[65] Hussen BM , Abdullah ST , Salihi A , et al. The emerging roles of NGS in clinical oncology and personalized medicine. Pathol Res Pract. 2022;230:153760. 35033746 10.1016/j.prp.2022.153760

[66] Morganti S , Tarantino P , Ferraro E , et al. Next generation sequencing (NGS): a revolutionary technology in pharmacogenomics and personalized medicine in cancer. Adv Exp Med Biol. 2019;1168:9‐30. 31713162 10.1007/978-3-030-24100-1_2

[67] Dong D , Shen G , Da Y , et al. Successful treatment of patients with refractory high‐grade serous ovarian cancer with GOPC‐ROS1 fusion using Crizotinib: a case report. Oncologist. 2020;25(11):e1720‐e1724. 32652753 10.1634/theoncologist.2019-0609 PMC7648329

[68] Aggarwal C , Thompson JC , Black TA , et al. Clinical implications of plasma‐based genotyping with the delivery of personalized therapy in metastatic non‐small cell lung cancer. JAMA Oncol. 2019;5(2):173‐180. 30325992 10.1001/jamaoncol.2018.4305 PMC6396811

[69] Tan AC , Lai GGY , Tan GS , et al. Utility of incorporating next‐generation sequencing (NGS) in an Asian non‐small cell lung cancer (NSCLC) population: incremental yield of actionable alterations and cost‐effectiveness analysis. Lung Cancer. 2020;139:207‐215. 31835042 10.1016/j.lungcan.2019.11.022

[70] Li W , Guo L , Liu Y , et al. Potential unreliability of uncommon ALK, ROS1, and RET genomic breakpoints in predicting the efficacy of targeted therapy in NSCLC. J Thorac Oncol. 2021;16(3):404‐418. 33248323 10.1016/j.jtho.2020.10.156

[71] Benayed R , Offin M , Mullaney K , et al. High yield of RNA sequencing for targetable kinase fusions in lung adenocarcinomas with no mitogenic driver alteration detected by DNA sequencing and low tumor mutation burden. Clin Cancer Res. 2019;25(15):4712‐4722. 31028088 10.1158/1078-0432.CCR-19-0225 PMC6679790

[72] Lovly CM , Heuckmann JM , de Stanchina E , et al. Insights into ALK‐driven cancers revealed through development of novel ALK tyrosine kinase inhibitors. Cancer Res. 2011;71(14):4920‐4931. 21613408 10.1158/0008-5472.CAN-10-3879 PMC3138877

[73] Roys A , Chang X , Liu Y , Xu X , Wu Y , Zuo D . Resistance mechanisms and potent‐targeted therapies of ROS1‐positive lung cancer. Cancer Chemother Pharmacol. 2019;84(4):679‐688. 31256210 10.1007/s00280-019-03902-6

[74] Kato Y , Ninomiya K , Ohashi K , et al. Combined effect of cabozantinib and gefitinib in crizotinib‐resistant lung tumors harboring ROS1 fusions. Cancer Sci. 2018;109(10):3149‐3158. 30053332 10.1111/cas.13752 PMC6172052

[75] Papadopoulos KP , Borazanci E , Shaw AT , et al. U.S. phase I first‐in‐human study of Taletrectinib (DS‐6051b/AB‐106), a ROS1/TRK inhibitor, in patients with advanced solid tumors. Clin Cancer Res. 2020;26(18):4785‐4794. 32591465 10.1158/1078-0432.CCR-20-1630

[76] Yun MR , Kim DH , Kim SY , et al. Repotrectinib exhibits potent antitumor activity in treatment‐naive and solvent‐front‐mutant ROS1‐rearranged non‐small cell lung cancer. Clin Cancer Res. 2020;26(13):3287‐3295. 32269053 10.1158/1078-0432.CCR-19-2777 PMC10283448

[77] Davare MA , Vellore NA , Wagner JP , et al. Structural insight into selectivity and resistance profiles of ROS1 tyrosine kinase inhibitors. Proc Natl Acad Sci USA. 2015;112(39):E5381‐E5390. 26372962 10.1073/pnas.1515281112 PMC4593101

[78] Tian Y , Yu Y , Shen Y , et al. Molecular simulation studies on the binding selectivity of type‐I inhibitors in the complexes with ROS1 versus ALK. J Chem Inf Model. 2017;57(4):977‐987. 28318251 10.1021/acs.jcim.7b00019

[79] Keddy C , Shinde P , Jones K , et al. Resistance Profile and structural modeling of next‐generation ROS1 tyrosine kinase inhibitors. Mol Cancer Ther. 2022;21(2):336‐346. 34907086 10.1158/1535-7163.MCT-21-0395 PMC8828706

[80] Lin JJ , Choudhury NJ , Yoda S , et al. Spectrum of mechanisms of resistance to Crizotinib and Lorlatinib in ROS1 fusion‐positive lung cancer. Clin Cancer Res. 2021;27(10):2899‐2909. 33685866 10.1158/1078-0432.CCR-21-0032 PMC8127383

[81] Inoue M , Toki H , Matsui J , et al. Mouse models for ROS1‐fusion‐positive lung cancers and their application to the analysis of multikinase inhibitor efficiency. Carcinogenesis. 2016;37(5):452‐460. 26964870 10.1093/carcin/bgw028

[82] Li Y , Chen X , Qu Y , et al. Partial response to ceritinib in a patient with abdominal inflammatory Myofibroblastic tumor carrying a TFG‐ROS1 fusion. J Natl Compr Cancer Netw. 2019;17(12):1459‐1462. 10.6004/jnccn.2019.7360 31805529

[83] Drilon A , Ou SI , Cho BC , et al. Repotrectinib (TPX‐0005) is a next‐generation ROS1/TRK/ALK inhibitor that potently inhibits ROS1/TRK/ALK solvent‐ front mutations. Cancer Discov. 2018;8(10):1227‐1236. 30093503 10.1158/2159-8290.CD-18-0484

[84] Davare MA , Saborowski A , Eide CA , et al. Foretinib is a potent inhibitor of oncogenic ROS1 fusion proteins. Proc Natl Acad Sci USA. 2013;110(48):19519‐19524. 24218589 10.1073/pnas.1319583110 PMC3845150

[85] Facchinetti F , Loriot Y , Kuo MS , et al. Crizotinib‐resistant ROS1 mutations reveal a predictive kinase inhibitor sensitivity model for ROS1‐ and ALK‐rearranged lung cancers. Clin Cancer Res. 2016;22(24):5983‐5991. 27401242 10.1158/1078-0432.CCR-16-0917

[86] Rodak O , Peris‐Diaz MD , Olbromski M , et al. Current landscape of non‐small cell lung cancer: epidemiology, histological classification, targeted therapies, and immunotherapy. Cancers (Basel). 2021;13(18):4705. 34572931 10.3390/cancers13184705 PMC8470525

[87] Yu ZQ , Wang M , Zhou W , et al. ROS1‐positive non‐small cell lung cancer (NSCLC): biology, diagnostics, therapeutics and resistance. J Drug Target. 2022;30(8):845‐857. 35658765 10.1080/1061186X.2022.2085730

[88] Shaw AT , Ou SH , Bang YJ , et al. Crizotinib in ROS1‐rearranged non‐small‐cell lung cancer. N Engl J Med. 2014;371(21):1963‐1971. 25264305 10.1056/NEJMoa1406766 PMC4264527

[89] Shaw AT , Riely GJ , Bang YJ , et al. Crizotinib in ROS1‐rearranged advanced non‐small‐cell lung cancer (NSCLC): updated results, including overall survival, from PROFILE 1001. Ann Oncol. 2019;30(7):1121‐1126. 30980071 10.1093/annonc/mdz131 PMC6637370

[90] Mazieres J , Zalcman G , Crino L , et al. Crizotinib therapy for advanced lung adenocarcinoma and a ROS1 rearrangement: results from the EUROS1 cohort. J Clin Oncol. 2015;33(9):992‐999. 25667280 10.1200/JCO.2014.58.3302

[91] Michels S , Massutí B , Schildhaus HU , et al. Safety and efficacy of Crizotinib in patients with advanced or metastatic ROS1‐rearranged lung cancer (EUCROSS): a European phase II clinical trial. J Thorac Oncol. 2019;14(7):1266‐1276. 30978502 10.1016/j.jtho.2019.03.020

[92] Patil T , Simons E , Mushtaq R , et al. Targeted therapies for ROS1‐rearranged non‐small cell lung cancer. Drugs Today (Barc). 2019;55(10):641‐652. 31720561 10.1358/dot.2019.55.10.3030646

[93] Friboulet L , Li N , Katayama R , et al. The ALK inhibitor ceritinib overcomes crizotinib resistance in non‐small cell lung cancer. Cancer Discov. 2014;4(6):662‐673. 24675041 10.1158/2159-8290.CD-13-0846 PMC4068971

[94] Lim SM , Kim HR , Lee JS , et al. Open‐label, multicenter, phase II study of ceritinib in patients with non‐small‐cell lung cancer harboring ROS1 rearrangement. J Clin Oncol. 2017;35(23):2613‐2618. 28520527 10.1200/JCO.2016.71.3701

[95] Morris TA , Khoo C , Solomon BJ . Targeting ROS1 rearrangements in non‐small cell lung cancer: Crizotinib and newer generation tyrosine kinase inhibitors. Drugs. 2019;79(12):1277‐1286. 31313100 10.1007/s40265-019-01164-3

[96] Yue D , Qian J , Chen Z , et al. Short‐term response to immune‐chemotherapy and immune features of a ceritinib‐resistant patient with ROS1‐rearranged lung adenocarcinoma. J Immunother Cancer. 2021;9(2):e001967. 33558279 10.1136/jitc-2020-001967 PMC7871696

[97] Shaw AT , Kim DW , Mehra R , et al. Ceritinib in ALK‐rearranged non‐small‐cell lung cancer. N Engl J Med. 2014;370(13):1189‐1197. 24670165 10.1056/NEJMoa1311107 PMC4079055

[98] Shaw AT , Kim TM , Crino L , et al. Ceritinib versus chemotherapy in patients with ALK‐rearranged non‐small‐cell lung cancer previously given chemotherapy and crizotinib (ASCEND‐5): a randomised, controlled, open‐label, phase 3 trial. Lancet Oncol. 2017;18(7):874‐886. 28602779 10.1016/S1470-2045(17)30339-X

[99] Rolfo C , Ruiz R , Giovannetti E , et al. Entrectinib: a potent new TRK, ROS1, and ALK inhibitor. Expert Opin Investig Drugs. 2015;24(11):1493‐1500. 10.1517/13543784.2015.1096344 26457764

[100] Drilon A , Siena S , Dziadziuszko R , et al. Entrectinib in ROS1 fusion‐positive non‐small‐cell lung cancer: integrated analysis of three phase 1‐2 trials. Lancet Oncol. 2020;21(2):261‐270. 31838015 10.1016/S1470-2045(19)30690-4 PMC7811790

[101] Dziadziuszko R , Krebs MG , De Braud F , et al. Updated integrated analysis of the efficacy and safety of entrectinib in locally advanced or metastatic ROS1 fusion‐positive non‐small‐cell lung cancer. J Clin Oncol. 2021;39(11):1253‐1263. 33646820 10.1200/JCO.20.03025 PMC8078299

[102] Fischer H , Ullah M , de la Cruz CC , et al. Entrectinib, a TRK/ROS1 inhibitor with anti‐CNS tumor activity: differentiation from other inhibitors in its class due to weak interaction with P‐glycoprotein. Neuro‐Oncology. 2020;22(6):819‐829. 32383735 10.1093/neuonc/noaa052 PMC7283026

[103] Sehgal K , Piper‐Vallillo AJ , Viray H , et al. Cases of ROS1‐rearranged lung cancer: when to use crizotinib, entrectinib, lorlatinib, and beyond? Precis Cancer Med. 2020;3:17. 32776005 10.21037/pcm-2020-potb-02 PMC7410006

[104] Frampton JE . Entrectinib: a review in NTRK+ solid tumours and ROS1+NSCLC. Drugs. 2021;81(6):697‐708. 33871816 10.1007/s40265-021-01503-3 PMC8149347

[105] Chong CR , Bahcall M , Capelletti M , et al. Identification of existing drugs that effectively target NTRK1 and ROS1 rearrangements in lung cancer. Clin Cancer Res. 2017;23(1):204‐213. 27370605 10.1158/1078-0432.CCR-15-1601 PMC5203969

[106] Shaw AT , Solomon BJ , Chiari R , et al. Lorlatinib in advanced ROS1‐positive non‐small‐cell lung cancer: a multicentre, open‐label, single‐arm, phase 1‐2 trial. Lancet Oncol. 2019;20(12):1691‐1701. 31669155 10.1016/S1470-2045(19)30655-2

[107] Drilon A , Camidge DR , Lin JJ , et al. Repotrectinib in ROS1 fusion‐positive non‐small‐cell lung cancer. N Engl J Med. 2024;390(2):118‐131. 38197815 10.1056/NEJMoa2302299 PMC11702311

[108] Ou SI , Fujiwara Y , Shaw AT , et al. Efficacy of taletrectinib (AB‐106/DS‐6051b) in ROS1+ NSCLC: an updated pooled analysis of U.S. and Japan phase 1 studies. JTO Clin Res Rep. 2021;2(1):100108. 34589973 10.1016/j.jtocrr.2020.100108 PMC8474193

[109] Ma Y , Zhao H , Xue J , et al. First‐in‐human phase I study of TQ‐B3139 (CT‐711) in advanced non‐small cell lung cancer patients with ALK and ROS1 rearrangements. Eur J Cancer. 2022;173:238‐249. 35940055 10.1016/j.ejca.2022.06.037

[110] Sun TY , Niu X , Chakraborty A , Neal JW , Wakelee HA . Lengthy progression‐free survival and intracranial activity of Cabozantinib in patients with crizotinib and ceritinib‐resistant ROS1‐positive non‐small cell lung cancer. J Thorac Oncol. 2019;14(2):e21‐e24. 30217491 10.1016/j.jtho.2018.08.2030

[111] Dudnik E , Agbarya A , Grinberg R , et al. Clinical activity of brigatinib in ROS1‐rearranged non‐small cell lung cancer. Clin Transl Oncol. 2020;22(12):2303‐2311. 32462394 10.1007/s12094-020-02376-w

[112] Solomon BJ , Besse B , Bauer TM , et al. Lorlatinib in patients with ALK‐positive non‐small‐cell lung cancer: results from a global phase 2 study. Lancet Oncol. 2018;19(12):1654‐1667. 30413378 10.1016/S1470-2045(18)30649-1

[113] Katayama R , Gong B , Togashi N , et al. The new‐generation selective ROS1/NTRK inhibitor DS‐6051b overcomes crizotinib resistant ROS1‐G2032R mutation in preclinical models. Nat Commun. 2019;10(1):3604. 31399568 10.1038/s41467-019-11496-z PMC6688997

[114] Katayama R , Kobayashi Y , Friboulet L , et al. Cabozantinib overcomes crizotinib resistance in ROS1 fusion‐positive cancer. Clin Cancer Res. 2015;21(1):166‐174. 25351743 10.1158/1078-0432.CCR-14-1385 PMC4286456

[115] Hegde A , Hong DS , Behrang A , et al. Activity of brigatinib in crizotinib and ceritinib‐resistant ROS1‐rearranged non‐small‐cell lung cancer. JCO Precis Oncol. 2019;3:1‐6. 10.1200/PO.18.00267 PMC7410165 32775947

[116] Choudhury NJ , Schneider JL , Patil T , et al. Response to immune checkpoint inhibition as monotherapy or in combination with chemotherapy in metastatic ROS1‐rearranged lung cancers. JTO Clin Res Rep. 2021;2(7):100187. 34590036 10.1016/j.jtocrr.2021.100187 PMC8474494

[117] Guisier F , Dubos‐Arvis C , Viñas F , et al. Efficacy and safety of anti‐PD‐1 immunotherapy in patients with advanced NSCLC with BRAF, HER2, or MET mutations or RET translocation: GFPC 01‐2018. J Thorac Oncol. 2020;15(4):628‐636. 31945494 10.1016/j.jtho.2019.12.129

[118] Mazieres J , Drilon A , Lusque A , et al. Immune checkpoint inhibitors for patients with advanced lung cancer and oncogenic driver alterations: results from the IMMUNOTARGET registry. Ann Oncol. 2019;30(8):1321‐1328. 31125062 10.1093/annonc/mdz167 PMC7389252

[119] Bylicki O , Tomasini P , Radj G , et al. Atezolizumab with or without bevacizumab and platinum‐pemetrexed in patients with stage IIIB/IV non‐squamous non‐small cell lung cancer with EGFR mutation, ALK rearrangement or ROS1 fusion progressing after targeted therapies: a multicentre phase II open‐label non‐randomised study GFPC 06‐2018. Eur J Cancer. 2023;183:38‐48. 36801605 10.1016/j.ejca.2023.01.014

[120] Comandini D , Catalano F , Grassi M , et al. Outstanding response in a patient with ROS1‐rearranged inflammatory myofibroblastic tumor of soft tissues treated with Crizotinib: case report. Front Oncol. 2021;11:658327. 34211840 10.3389/fonc.2021.658327 PMC8239351

[121] Robertson SJ , Orme L , Teixeira R , et al. Evaluation of Crizotinib treatment in a patient with unresectable GOPC‐ROS1 fusion agminated Spitz nevi. JAMA Dermatol. 2021;157(7):836‐841. 34076666 10.1001/jamadermatol.2021.0025 PMC8173474

[122] Jakubowski CD , Mohan AA , Kamel IR , Yarchoan M . Response to Crizotinib in ROS1 fusion‐positive intrahepatic Cholangiocarcinoma. JCO Precis Oncol. 2020;4:825‐828. 35050759 10.1200/PO.20.00116 PMC9797238

[123] Li J , Liu L , Zhang Q , et al. A novel TJP1‐ROS1 fusion in malignant peripheral nerve sheath tumor responding to crizotinib: a case report. Medicine (Baltimore). 2020;99(26):e20725. 32590748 10.1097/MD.0000000000020725 PMC7328986

[124] Mayr L , Guntner AS , Madlener S , et al. Cerebrospinal fluid penetration and combination therapy of entrectinib for disseminated ROS1/NTRK‐fusion positive pediatric high‐grade glioma. J Pers Med. 2020;10(4):290. 33353026 10.3390/jpm10040290 PMC7766483

[125] Papusha L , Zaytseva M , Panferova A , et al. Two clinically distinct cases of infant hemispheric glioma carrying ZCCHC8:ROS1 fusion and responding to entrectinib. Neuro‐Oncology. 2022;24(6):1029‐1031. 35196386 10.1093/neuonc/noac026 PMC9159448

[126] Couts KL , McCoach CE , Murphy D , et al. Acral lentiginous melanoma harboring a ROS1 gene fusion with clinical response to entrectinib. JCO Precis Oncol. 2017;1:1‐7. 10.1200/PO.16.00013 35172482

[127] Pishvaian MJ , Garrido‐Laguna I , Liu SV , Multani PS , Chow‐Maneval E , Rolfo C . Entrectinib in TRK and ROS1 fusion‐positive metastatic pancreatic cancer. JCO Precis Oncol. 2018;2:1‐7. 10.1200/PO.18.00039 35135135

[128] Velthaus JL , Iglauer P , Simon R , et al. Lorlatinib induces durable disease stabilization in a pancreatic cancer patient with a ROS1 p.L1950F mutation: case report. Oncol Res Treat. 2021;44(9):495‐502. 34320493 10.1159/000517616

[129] Carcamo B , Bista R , Wilson H , Reddy P , Pacheco J . Rapid response to lorlatinib in a patient with TFG‐ROS1 fusion positive inflammatory myofibroblastic tumor of the chest wall metastatic to the brain and refractory to first and second generation ROS1 inhibitors. J Pediatr Hematol Oncol. 2021;43(5):e718‐e722. 34157012 10.1097/MPH.0000000000002185

[130] Spigel DR , Reynolds C , Waterhouse D , et al. Phase 1/2 study of the safety and tolerability of Nivolumab plus Crizotinib for the first‐line treatment of anaplastic lymphoma kinase translocation ‐ positive advanced non‐small cell lung cancer (CheckMate 370). J Thorac Oncol. 2018;13(5):682‐688. 29518553 10.1016/j.jtho.2018.02.022

[131] Bauer TM , Felip E , Solomon BJ , et al. Clinical management of adverse events associated with lorlatinib. Oncologist. 2019;24(8):1103‐1110. 30890623 10.1634/theoncologist.2018-0380 PMC6693708

[132] Cocco E , Scaltriti M , Drilon A . NTRK fusion‐positive cancers and TRK inhibitor therapy. Nat Rev Clin Oncol. 2018;15(12):731‐747. 30333516 10.1038/s41571-018-0113-0 PMC6419506

[133] Drilon A , Clark JW , Weiss J , et al. Antitumor activity of crizotinib in lung cancers harboring a MET exon 14 alteration. Nat Med. 2020;26(1):47‐51. 31932802 10.1038/s41591-019-0716-8 PMC8500676

[134] Camidge DR , Otterson GA , Clark JW , et al. Crizotinib in patients with MET‐amplified NSCLC. J Thorac Oncol. 2021;16(6):1017‐1029. 33676017 10.1016/j.jtho.2021.02.010

[135] Lin JJ , Chin E , Yeap BY , et al. Increased hepatotoxicity associated with sequential immune checkpoint inhibitor and Crizotinib therapy in patients with non‐small cell lung cancer. J Thorac Oncol. 2019;14(1):135‐140. 30205166 10.1016/j.jtho.2018.09.001 PMC6309637

[136] Fortes BH , Tailor PD , Dalvin LA . Ocular toxicity of targeted anticancer agents. Drugs. 2021;81(7):771‐823. 33788182 10.1007/s40265-021-01507-z

[137] Li YX , Yang JY , Xu YF , et al. A meta‐analysis of the comparing of the first‐generation and next‐generation TKIs in the treatment of NSCLC. Math Biosci Eng. 2019;16(5):5687‐5696. 31499732 10.3934/mbe.2019283

[138] Zhang L , Ren HW , Wu QL , Wu YJ , Song X . The effect of next‐generation TKI in non‐small cell lung cancer after failure of first‐line treatment: a meta‐analysis. Pathol Oncol Res. 2020;26(2):1137‐1143. 31147837 10.1007/s12253-019-00669-2

[139] McCoach CE , Le AT , Gowan K , et al. Resistance mechanisms to targeted therapies in ROS1(+) and ALK(+) non‐small cell lung cancer. Clin Cancer Res. 2018;24(14):3334‐3347. 29636358 10.1158/1078-0432.CCR-17-2452 PMC6050099

[140] Vaishnavi A , Schubert L , Rix U , et al. EGFR mediates responses to small‐molecule drugs targeting oncogenic fusion kinases. Cancer Res. 2017;77(13):3551‐3563. 28428274 10.1158/0008-5472.CAN-17-0109 PMC5516930

[141] Guaitoli G , Bertolini F , Bettelli S , et al. Deepening the knowledge of ROS1 rearrangements in non‐small cell lung cancer: diagnosis, treatment, resistance and concomitant alterations. Int J Mol Sci. 2021;22(23):12867. 34884672 10.3390/ijms222312867 PMC8657497

[142] Gainor JF , Tseng D , Yoda S , et al. Patterns of metastatic spread and mechanisms of resistance to Crizotinib in ROS1‐positive non‐small‐cell lung cancer. JCO Precis Oncol. 2017;2017:1‐13. 10.1200/PO.17.00063 PMC5766287 29333528

[143] Song A , Kim TM , Kim DW , et al. Molecular changes associated with acquired resistance to Crizotinib in ROS1‐rearranged non‐small cell lung cancer. Clin Cancer Res. 2015;21(10):2379‐2387. 25688157 10.1158/1078-0432.CCR-14-1350

[144] Awad MM , Engelman JA , Shaw AT . Acquired resistance to crizotinib from a mutation in CD74‐ROS1. N Engl J Med. 2013;369(12):1173. 10.1056/NEJMc1309091 24047072

[145] Drilon A , Somwar R , Wagner JP , et al. A novel Crizotinib‐resistant solvent‐front mutation responsive to Cabozantinib therapy in a patient with ROS1‐rearranged lung cancer. Clin Cancer Res. 2016;22(10):2351‐2358. 26673800 10.1158/1078-0432.CCR-15-2013 PMC4867287

[146] Wu X , Fu Y , Wang Y , Wan SH , Zhang JJ . Gaining insight into crizotinib resistance mechanisms caused by L2026M and G2032R mutations in ROS1 via molecular dynamics simulations and free‐energy calculations. J Mol Model. 2017;23(4):141. 28361443 10.1007/s00894-017-3314-z

[147] Sasaki T , Koivunen J , Ogino A , et al. A novel ALK secondary mutation and EGFR signaling cause resistance to ALK kinase inhibitors. Cancer Res. 2011;71(18):6051‐6060. 21791641 10.1158/0008-5472.CAN-11-1340 PMC3278914

[148] Doebele RC , Pilling AB , Aisner DL , et al. Mechanisms of resistance to crizotinib in patients with ALK gene rearranged non‐small cell lung cancer. Clin Cancer Res. 2012;18(5):1472‐1482. 22235099 10.1158/1078-0432.CCR-11-2906 PMC3311875

[149] Dziadziuszko R , Le AT , Wrona A , et al. An activating KIT mutation induces Crizotinib resistance in ROS1‐positive lung cancer. J Thorac Oncol. 2016;11(8):1273‐1281. 27068398 10.1016/j.jtho.2016.04.001 PMC4961521

[150] Yang X , Tang Z , Li J , Jiang J , Liu Y . Progress of non‐small‐cell lung cancer with ROS1 rearrangement. Front Mol Biosci. 2023;10:1238093. 38187090 10.3389/fmolb.2023.1238093 PMC10766828

[151] Lin JJ , Langenbucher A , Gupta P , et al. Small cell transformation of ROS1 fusion‐positive lung cancer resistant to ROS1 inhibition. NPJ Precis Oncol. 2020;4:21. 32802958 10.1038/s41698-020-0127-9 PMC7400592

[152] Killock D . Lorlatinib in ROS1‐positive NSCLC. Nat Rev Clin Oncol. 2020;17(1):7. 10.1038/s41571-019-0301-6 31705129

[153] Davies KD , Mahale S , Astling DP , et al. Resistance to ROS1 inhibition mediated by EGFR pathway activation in non‐small cell lung cancer. PLoS One. 2013;8(12):e82236. 24349229 10.1371/journal.pone.0082236 PMC3862576

[154] Patil T , Smith DE , Bunn PA , et al. The incidence of brain metastases in stage IV ROS1‐rearranged non‐small cell lung cancer and rate of central nervous system progression on Crizotinib. J Thorac Oncol. 2018;13(11):1717‐1726. 29981925 10.1016/j.jtho.2018.07.001 PMC6204290

[155] Li Z , Shen L , Ding D , et al. Efficacy of Crizotinib among different types of ROS1 fusion partners in patients with ROS1‐rearranged non‐small cell lung cancer. J Thorac Oncol. 2018;13(7):987‐995. 29704675 10.1016/j.jtho.2018.04.016

[156] Jun HJ , Johnson H , Bronson RT , de Feraudy S , White F , Charest A . The oncogenic lung cancer fusion kinase CD74‐ROS activates a novel invasiveness pathway through E‐Syt1 phosphorylation. Cancer Res. 2012;72(15):3764‐3774. 22659450 10.1158/0008-5472.CAN-11-3990 PMC3753671

[157] Landi L , Cappuzzo F . How selecting best upfront therapy for metastatic disease?‐focus on ROS1‐rearranged disease. Transl Lung Cancer Res. 2020;9(6):2686‐2695. 33489827 10.21037/tlcr-20-1109 PMC7815342

